# Extreme Clonality in Lymphoblastoid Cell Lines with Implications for Allele Specific Expression Analyses

**DOI:** 10.1371/journal.pone.0002966

**Published:** 2008-08-13

**Authors:** Vincent Plagnol, Elif Uz, Chris Wallace, Helen Stevens, David Clayton, Tayfun Ozcelik, John A. Todd

**Affiliations:** 1 JDRF/WT Diabetes and Inflammation Laboratory, University of Cambridge, Cambridge, United Kingdom; 2 Department of Molecular Biology and Genetics, Faculty of Science, Bilkent University, Ankara, Turkey; 3 Clinical Pharmacology, William Harvey Research Institute, Bart's and The London, London, United Kingdom; 4 Institute of Materials Science and Nanotechnology (UNAM), Bilkent University, Ankara, Turkey; Texas Tech University Health Sciences Center, United States of America

## Abstract

Lymphoblastoid cell lines (LCL) are being actively and extensively used to examine the expression of specific genes and genome-wide expression profiles, including allele specific expression assays. However, it has recently been shown that approximately 10% of human genes exhibit random patterns of monoallelic expression within single clones of LCLs. Consequently allelic imbalance studies could be significantly compromised if bulk populations of donor cells are clonal, or near clonal. Here, using X chromosome inactivation as a readout, we confirm and quantify widespread near monoclonality in two independent sets of cell lines. Consequently, we recommend where possible the use of bulk, non cell line, *ex vivo* cells for allele specific expression assays.

## Introduction

Lymphoblastoid cell lines (LCL), which have been immortalised by infection with Epstein Barr Virus (EBV), are being actively and extensively used to examine the expression of specific genes and genome-wide expression profiles [1], [2], [3], [4]. Researchers are linking and associating single nucleotide polymorphisms (SNPs) with inherited, expression quantitative trait loci (eQTL) using tens to hundreds of LCLs. A complementary approach is the analysis of allelic imbalance of gene expression owing to unequal transcription (or splicing) from the two alleles or haplotypes using RNA samples from individuals who are heterozygous at the eQTL SNP. Allelic imbalance approaches have the advantage of assessing expression within an individual rather than across subjects thereby avoiding several sources of error and variation. However, it has recently been shown that approximately 10% of human genes exhibit random patterns of monoallelic expression within single clones of LCLs [5]. Consequently, in a clonal or near clonal LCL, gene expression measurements may not be representative of the *in vivo* cell population and allelic imbalance studies could be significantly compromised.

Nevertheless, although it is published [6] that some widely used LCLs are pauciclonal or even monoclonal, it is still not fully appreciated that bulk LCL cultures can be highly restricted in the number of constituent clones, as evidenced by the exclusive use of cell lines in most recent studies [1], [2], [3], [4]. Here, using X chromosome inactivation (XCI) as a measure of the degree of clonality, we confirm and quantify widespread near monoclonality in two independent sets of 466 and 708 cell lines. Our results suggest that the loss of diversity occurs in the early stages of the LCL preparation and, therefore, affects equally freshly prepared, as well as established cell lines such as the HapMap LCLs [6].

## Results

**Figure 1:**
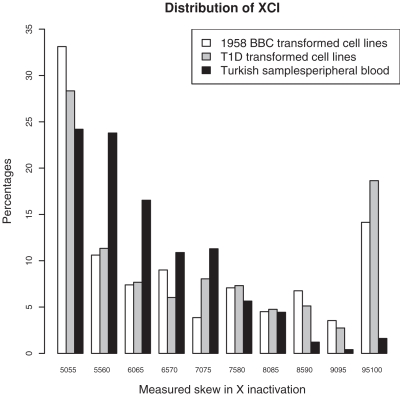
Distribution of XCI in the British 1958 Birth Cohort samples, JDRF/WT T1D cases collection (both with DNA extracted from transformed cells lines) and the control Turkish population (DNA extracted from peripheral blood).

We measured XCI using a standard assay in all our samples [7] and observed large differences between cell lines (T1D and British 1958 Birth Cohort [8]) and controls (healthy Turkish women) for which DNA was isolated directly from peripheral blood (Figure 1). This high skew in XCI is associated with clonality in LCLs: when a LCL reaches near clonality, the skew in XCI tends to increase until reaching 100% [9]. We used these differences between both sets of healthy samples (British 1958 Birth Cohort cell lines and Turkish controls) to quantify the reduction in diversity in the transformed cell lines.

Our statistical model assumes two potential outcomes for a cell line transformation. In the first case, with probability 1-*f*, the transformed cell line accurately reflects the level of skewing from the initial cell population. In the second case, with probability *f*, the transformation process subsamples *n* cells from the initial pool and the final population consists of an equal mixture of the descendants of these *n* cells. Note that we do not assume that the final cell line is formed from only the descendants of *n* cells, but that the combination of the initial LCL transformation with the variation in growth rate among cell lineages leads to a bias in measurement equivalent to a bottleneck of *n* cells which then grow equally. We present this estimate as an informative summary statistic of the effect of near clonality on the expression measurement. We assume that the number *n* is distributed as a Poisson random variable with mean μ and we are interested in the joint estimation of both parameters *f* and μ.

**Figure 2:**
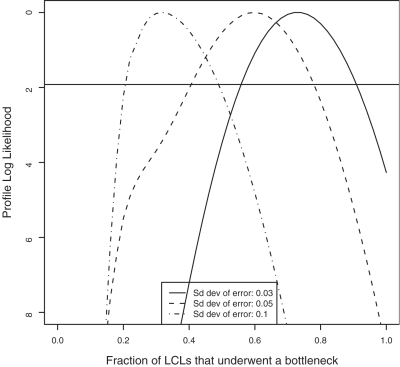
Likelihood curve for the fraction of cells *f* that underwent a bottleneck. We considered three values for the standard error in the measurement of the skew in X inactivation (standard deviation of 0.03, 0.05 and 0.1). The horizontal line indicates the 95% confidence interval.

We first computed the profile log-likelihood for the parameter *f* denoting the fraction of cell lines that underwent a bottleneck (see Figure 2, Data S1 and Code S1). We found that the maximum likelihood estimate for *f* varies with the accuracy of the XCI assay, this estimate going down when the average error increases (see Figure 2). The precision of the XCI assay is not known exactly but the average error is expected to lie within 0.03–0.05 [10]. Assuming a XCI assay average error of 0.05, we estimated that pauciclonality affects 60% of the LCLs. When the XCI assay error varied between 0.03 and 0.05, the average number of clones in pauciclonal LCLs was estimated between 4 and 5.

However, while previous estimates suggest that an average error of 0.1 for the XCI is an overestimation, this scenario appeared to fit the data better (2Δlog*L* = 6.6, *p* = 0.01 compared to the best scenario assuming an average measurement error of 0.05). Moreover, assuming an average measurement error of 0.1, we cannot reject the assumption that the bottleneck always involves a single clone. It indicates that a likely scenario is a situation where the XCI in most LCLs reflect the XCI in whole blood but approximately 22% of the LCLs do not grow properly and become monoclonal.

These estimates rely on the assumption that the pattern of XCI is similar between the British population and the Turkish control samples. Indeed, the pattern of XCI is relatively constant across populations (see [10], [11], [12], [13], [14], [15] and Table 1) and ethnic differences are unlikely to explain the strong differences we observe.

**Table 1:** Levels of X chromosome inactivation skewing in different groups of healthy and diseased individuals.

Population	η_total_	η_informative_	>90%	80–89%	50–79%	Source of DNA
**T1D cases-Great Britain**						
T1D-≤40days transformation†		367	66 (18)	39 (10.4)	262 (71.6)	Cell line
T1D->40days transformation†		180	70 (38.9)	16 (8.9)	94 (52.2)	Cell line
T1D-all†	708	547	136 (24.8)	55 (10.1)	356 (65.1)	Cell line
**Healthy controls**						
**Great Britain**						
British 1958 Birth Cohort†	466	311	65 (20.9)	32 (10.3)	214 (68.8)	Cell line
**Turkey**						
Adult [11]	160	124	3 (2.41)	7 (5.6)	114 (91.9)	Peripheral blood
Children†	92	72	2 (2.8)	6 (8.3)	64 (88.9)	Peripheral blood
Newborn†	91	52	2 (3.8)	2 (3.8)	48 (92.3)	Peripheral blood
**North America**						
Adult/Mix-US [10]	-	415	22 (5.3)	59 (14.2)	334 (80.5)	Peripheral blood
Adult/Unknown-US [14]	114	100	1 (1.0)	7 (7.0)	92 (92.0)	Peripheral blood
Newborn-USA [10]	-	590	4 (0.7)	29 (4.9)	557 (94.4)	Peripheral blood
Adult/Unknown-Canada [12]	109	97	8 (8.2)	15 (15.0)	74 (76.3)	Peripheral blood
**Other**						
Adult/Caucasian-Italy [13]	-	164	10 (6.1)	22 (13.4)	132 (80.5)	Peripheral blood
Adult/Caucasian-Denmark [15]	-	96	1 (1.0)	10 (10.0)	85 (89.0)	Peripheral blood
Adult/Caucasian-Tunisia†	97	46	4 (8.7)	5 (10.9)	37 (80.4)	Peripheral blood

The first number represents the number of samples in each of the three categories (XCI>90%; between 80–89% and 50–79%). The number in parenthesis is the percentage this category represents.

† Unpublished.

**Figure 3:**
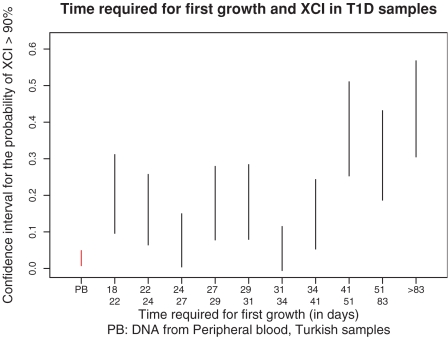
Confidence intervals for the probability of XCI>90% as a function of the time required for first growth (ie. between transformation and until the culture volume reaches 100 ml).

Using information about the cell line preparation for the T1D samples, we examined what variables explained the variability in XCI. Volume of blood drawn, date of bleed, age of blood at first freeze (before transformation) and number of re-growths (defined as successive cell line growths from a frozen sample) showed no significant correlation with XCI (*P*>0.05). However, the time required for first growth (defined as the time from transformation until the culture volume reaches 100 ml) is positively correlated with skewed XCI (correlation coefficient *ρ* = 0.19, *P* = 7×10^−6^). Figure 3 shows how extreme XCI (>90%) correlates with this covariate. These data suggest that loss of diversity occurs during or shortly after transformation: in the case of slow initial growth, stochastic variability would have an increased effect because of the small number of EBV infected cells. It is likely that subsequent events, including re-growths, have a limited impact because of a higher cell count when they occur. We also note that even cell lines with the shortest time for first growth (less than 22 days) are still significantly more skewed than our control samples (DNA obtained from whole blood), indicating that a robust early growth does not guarantee absence of clonality.

**Figure 4:**
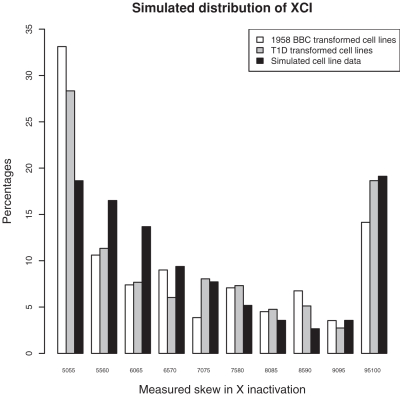
Simulation study comparing the XCI between our best fitting scenario and both sets of cell line (1958 British Birth Cohort and T1D samples).

We then investigated whether our statistical model could explain the pattern of XCI observed in the data. We simulated data using our best fitting parameters and compared the results with XCI in both sets of cell lines (Figure 4). This comparison provided mixed results. While our model properly explained the excess of samples with extreme skewing (95–100% XCI) observed in cell line samples, we could not explain the excess of cell lines samples with XCI between 50 and 55%. A potential explanation is that subtle differences occurred in the XCI assay. Because the XCI assay is primarily designed to identify highly skewed individuals, it is plausible that it is not robust to small experimental differences when trying to distinguish XCI in the 50–70% range.

## Discussion

Based on our XCI assay, we estimate that pauciclonality affects approximately 20% of the LCLs in our study. While XCI is a useful readout for pauciclonality, additional readouts, such as the number of tandem repeat sequences in the EBV genome [16] or the structure of the rearranged immunoglobulin heavy chain gene [17], might be useful in the future to confirm our estimates.

These results, combined with evidence of widespread random monoallelic expression [5], indicate that expression data from LCLs are not well suited to detect correlations between SNPs and gene expression. When for a given gene the expression is affected by methylation patterns or other epigenetic meiotically stable factors [5], the expression measurement in LCLs will not be representative of the *in vivo* cell population. Strong allelic imbalance can result from the random inactivation of the same allele in the small number of clones that constitute the LCL, resulting in increased false positive and false negative rates. Consequently, we expect that the fraction of human genes affected by monoallelic expression [5] will be highly differentially expressed in the approximately 20% of monoclonal LCLs. In fact, any gene expression measurement that is variable across cells *in vivo* can be significantly altered by the random subsampling of a small number of clones in a LCL. This additional measurement noise will affect the power of genome-wide association studies, or, indeed, specific gene studies to detect association between SNPs and expression traits in LCLs. Consequently, we recommend, where possible, to either screen the LCLs for monoclonality or use bulk, non cell line, *ex vivo* cells when measuring gene expression [18], and in particular when focusing on allele-specific expression [1].

## Methods

### Dataset

The data consisted of two sets of LCLs: 466 samples from healthy women (British 1958 Birth Cohort, see [8]) and 708 samples from type 1 diabetic women (Juvenile Diabetes Research Foundation/Wellcome Trust British T1D case collection), with all samples originating from England, Scotland and Wales. In addition, the control set consisted of 343 samples from Turkish healthy women for which DNA was isolated from peripheral blood. For the T1D cell lines, additional information describing the cell line preparation was available (see Protocol S1).

### X inactivation and clonality in LCL

X inactivation is a process by which, early in the female mammals' development, epigenetic modifications randomly inactivate one of the two copies of the X chromosome to guarantee a comparable gene dosage between male and females. Consequently, a female is a mosaic of two cell types in which either the maternal or the paternal chromosome is inactivated. The proportion of the most common of these two cell populations, expressed as a percentage between 50% and 100%, is called the level of skewing in XCI. We measured XCI using a standard assay [7].

### Mathematical model for XCI in cell lines

In the presence of a bottleneck, we modelled the skew in the cell line samples (denoted by the random variable *Y* ^*^) as follows:
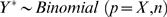
where *n*∼*Poisson*(μ) is the bottleneck size (we assume a Poisson random variable with mean μ that we want to estimate) and *X* is a random variable describing the skew in the population estimated from the Turkish control samples (using peripheral blood and not cell lines). We also investigated a version of this scenario where the bottleneck always involves a single clone. The XCI variable *Y*, measured between 0.5 and 1, is obtained by adding an error term ε:
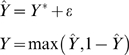
ε is a truncated Gaussian random variable with mean 0 and standard deviation σ = 0.03,0.05,0.1. The truncation ensures that *Yˆ*∈{0,1}.

### Likelihood estimation

The fraction of LCLs undergoing a bottleneck is denoted by *f* and the number of cells *n* in the bottleneck is *Poisson*(μ). Parameters are estimated using a maximum likelihood approach, maximizing the likelihood over a two dimensional grid of values for (*f*,μ). We summarized the XCI data using ten uniformly spaced bins 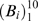. The distribution of the measured XCI, denoted by *Y*, is therefore multinomial with parameters (*p*_1_,…,*p*_10_) where *p_i_* = *P*(*Y*∈*B_i_*).

For given values of the parameters (*f*,μ) the probabilities *p_i_* are estimated as follows:
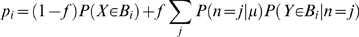
where *X* designates the XCI randomly sampled from the control Turkish population.

*P*(*Y*∈*B_i_*|*n* = *j*) is the probability that the measured skew *Y* is located in the bin *B_i_* conditionally on a bottleneck of size *j*:
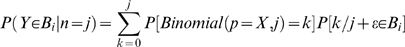



## Supporting Information

Data S1 XCI data for the three datasets studied in this paper
(0.01 MB XLS)

Code S1 R code (Sweave generated) used to generate figures and compute the loglikelihood profile.
(0.11 MB PDF)

Protocol S1 Protocol for cell line transformation.
(0.07 MB PDF)
