# Supplementary material for: Extreme Clonality in Lymphoblastoid Cell Lines with Implications for Allele Specific Expression Analyses
Source: PLoS One. 2008 Aug 13;3(8):e2966. doi: 10.1371/journal.pone.0002966 (PMC2494943; doi:10.1371/journal.pone.0002966)
Supplement: Code S1 — R code (Sweave generated) used to generate figures and compute the loglikelihood profile. (0.11 MB PDF) [file pone.0002966.s002.pdf]

Near clonality in lymphoblastoid cell lines and consequences on allele specific expression-R code for MLE estimation

Vincent Plagnol, Elif Uz, Chris Wallace, Helen Stevens,// David Clayton, Tayfun Ozcelik and John A. T

1 Distribution of XCI in cell lines

We first read the data and remove any missing values. We also set the bins we want to use to summarize the data and generate histograms for each of our three datasets: Turkish controls, 1958 British Birth Cohort, Type 1 Diabetes (T1D and 58C used cell line DNA). The output is shown in Figure 1.

```
> data <- read.csv("dataXinactivation.csv", header = TRUE)
> control <- data$Turkish.control[!is.na(data$Turkish.control)]
> T1D.data <- data$T1D[!is.na(data$T1D)]
> BBC58 <- data$BBC58[!is.na(data$BBC58)]
> bp <- c(50, 55, 60, 65, 70, 75, 80, 85, 90, 95, 100)
> names <- c("50-55", "55-60", "60-65", "65-70", "70-75", "75-80",
+     "80-85", "85-90", "90-95", "95-100")
> cont <- hist(control, plot = FALSE, breaks = bp)
> BBC <- hist(BBC58, plot = FALSE, breaks = bp)
> T1D <- hist(T1D.data, plot = FALSE, breaks = bp)
```

```
> pdf("fig/skew.pdf", height = 6, width = 7)
> barplot(rbind(100 * BBC$counts/sum(BBC$counts), 100 * T1D$counts/sum(T1D$counts),
+     100 * cont$counts/sum(cont$counts)), beside = TRUE, names = names,
+     cex.names = 0.85, ylim = c(0, 35), col = c("white", "grey",
+         "black"), ylab = "Percentages", xlab = "Measured skew in X inactivation",
+     cex.lab = 1.3)
> title("Distribution of XCI", cex.main = 1.5)
> legend(legend = c("1958 BBC transformed cell lines", "T1D transformed cell lines",
+     "Turkish samples-peripheral blood"), "topright", fill = c("white",
+     "grey", "black"), cex = 1.3)
> dev.off()

null device
          1

> cont$freq <- cont$counts/sum(cont$counts)
```

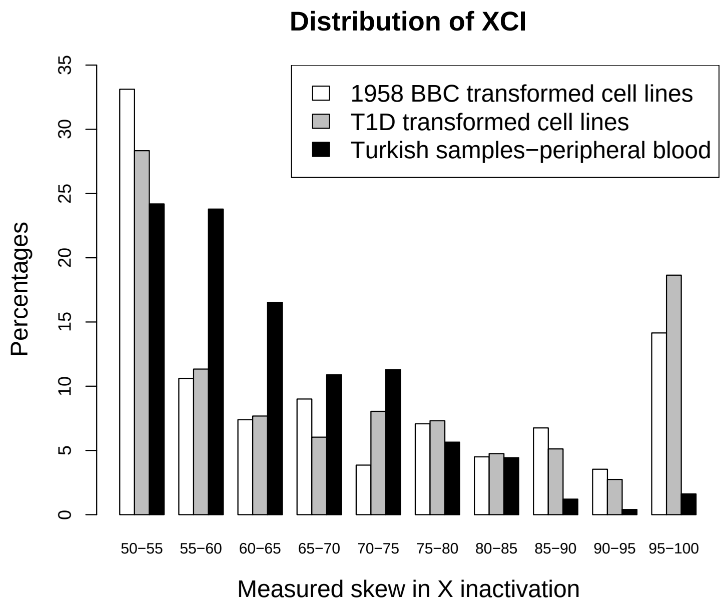

Figure 1: Distribution of XCI in the British 1958 Birth Cohort samples, JDRF/WT T1D cases collection (both with DNA extracted from transformed cells lines) and the control Turkish population (DNA extracted from peripheral blood).

2 Parameter estimation

2.1 Some notations

In the presence of a bottleneck, we modeled the skew in the cell line samples (denoted by the random variable Y^*) as follows:

$$Y^* \sim \text{Binomial}(p = X, n)$$

where $n \sim Poisson(\mu)$ is the bottleneck size (we assume a Poisson random variable with mean μ that we want to estimate). X is a random variable describing the skew in the population estimated from the Turkish control samples (using peripheral blood and not cell lines). The measured skew Y is obtained by adding an error term ϵ:

$$\hat{Y} = Y^* + \epsilon$$

$$Y = \max(\hat{Y}, 1 - \hat{Y})$$

ϵ is a Gaussian random variable with mean 0 and standard deviation $\sigma = 2, 5, 10\%$

2.2 Likelihood estimation

We divided the XCI data into ten uniformly spaced bins $(B_i)_1^{10}$. The fraction of LCLs undergoing a bottleneck is denoted by f and the number of cells n in the bottleneck is $Poisson(\mu)$. For given values of the parameters f and μ the expected relative proportions in each of these ten bins is estimated as follows:

$$\mathbb{P}(Y \in B_i) = (1 - f)\mathbb{P}(X \in B_i) + f \sum_j \mathbb{P}(n = j|\mu)\mathbb{P}(Y \in B_i|n = j)$$

$$\mathbb{P}(Y \in B_i|n = j) = \sum_{k=0}^{n} \mathbb{P}\left[Binomial(p = X, n) = k\right] \mathbb{P}(k/n + \epsilon \in B_i)$$

where X designates the XCI randomly sampled from the control Turkish population.

The difficult part of the estimation concernes the estimation of

$$\mathbb{P}(Y \in B_i|n = j)$$

We first need a simple routine to translate a continous XCI value in discrete bins. This routine also handles the fact that a XCI of 0.03 is counted as $1 - 0.03 = 0.97$ because our definition of the XCI implies that we measure the frequency of the largest population.

```
> profile.routine <- function(my.mean, error) {
+     q <- seq(0, 1, 0.05)
+     res <- diff(pnorm(mean = my.mean, sd = error, q = q))
+     res <- res/sum(res)
+     res <- rev(res[c(1:10)]) + res[11:20]
+     return(res)
+ }
```

We can now use this routine to estimate $\mathbb{P}(Y \in B_i|n = j)$. The result will be placed in a matrix with 40 rows and 10 columns. The columns correspond to the bins we are using. Each row correspond to a different value for the numbre of cells in the bottleneck. Note that the first step consists of sumamrizing the distribution of frequencies in the Turkish control population because we need to iterate over these background frequencies.

```
> list.skew <- as.numeric(names(table(control)))/100
> freq.skew <- as.vector(table(control))/length(control)
> ncontrols <- length(freq.skew)
> new.estimate.proba <- function(error) {
+     probabilities <- matrix(0, nrow = 40, ncol = 10)
+     for (i in c(1:ncontrols)) {
+         s.skew <- list.skew[i]
+         f.skew <- freq.skew[i]
+         for (bottleneck in c(1:40)) {
+             for (n in c(0:bottleneck)) {
+                 prob <- dbinom(x = n, size = bottleneck, p = s.skew)
+                 probabilities[bottleneck, ] <- probabilities[bottleneck,
+                   ] + f.skew * prob * profile.routine(n/bottleneck,
+                   error = error)
+             }
+         }
+     }
+     return(probabilities)
+ }
```

Another small routine we need is the distribution probabilities for a Poisson random variable conditionally on being greater than 0. Note the special case $\lambda = 0$ where we assume that the bottleneck always involves a single clone.

```
> my.new.density <- function(f, lambda) {
+     if (lambda == 0) {
+         poisson.dens <- rep(0, 40)
+         poisson.dens[1] <- 1
+     }
+     else {
+         poisson.dens <- dpois(lambda = lambda, x = c(1:40))
+         poisson.dens <- poisson.dens/sum(poisson.dens)
+     }
+     pr <- apply(poisson.dens * new.proba, MAR = 2, FUN = sum)
+     pr <- f * pr + (1 - f) * cont$freq
+     likelihood <- dmultinom(prob = pr, log = TRUE, x = BBC$counts)
+ }
```

We can now put it all together in the function `plot.profile` that evaluates the profile likelihood on a grid of values for f and μ.

```
> plot.profile <- function(err, j) {
+     f <- seq(0, 1, 0.02)
+     lambda <- c(0, seq(0.5, 7, 0.02))
+     new.proba <<- new.estimate.proba(error = err)
+     res <- matrix(f, ncol = 3, nrow = length(f))
+     for (i in c(1:length(f))) {
+         probabi <- sapply(lambda, FUN = function(x) {
+             my.new.density(f = res[i, 1], x)
+         })
+         res[i, 2] <- max(probabi)
+         res[i, 3] <- lambda[which.max(probabi)]
+     }
```

```
+     best.scenario <- which.max(res[, 2])
+     cat("The best scenario of an error rate of", err, "is:\n")
+     print(res[best.scenario, ])
+     lines(res[, 1], res[, 2] - max(res[, 2]), type = "l", lty = j)
+ }
```

And we eventually run the profile likelihood estimaiton in the following piece of code. The resuls are shown in Figure 2.

```
> error <- c(0.03, 0.05, 0.1)
> pdf("fig/f_profile.pdf")
> par(cex.lab = 1.3, cex.main = 1.5)
> plot(xlab = "Fraction of LCLs that underwent a bottleneck", ylab = "Profile Log Likelihood",
+     xlim = c(0, 1), ylim = c(-8, 0), x = NA, y = NA)
> my.lty <- c(1, 2, 4)
> for (j in c(1, 2, 3)) {
+     plot.profile(error[j], my.lty[j])
+ }
```

```
The best scenario of an error rate of 0.03 is:
[1]   0.72000 -61.22184   5.06000
The best scenario of an error rate of 0.05 is:
[1]   0.600 -60.878   3.960
The best scenario of an error rate of 0.1 is:
[1]   0.32000 -58.40938   0.98000
```

```
> legend(legend = paste("Sd dev of error:", error), lty = c(1,
+     2, 4), "bottomleft", inset = c(0.33, 0))
> abline(h = -qchisq(df = 1, p = 0.95)/2)
> dev.off()
```

```
null device
          1
```

3 Comparing simulation and data

We can now compare our best fitting scenario with the cell line data. With a 5% error rate estimated f is 52% and the average number of cells in the bottleneck is 4. The simulation function takes as an input these two parameters.

```
> simulate <- function(freq, bot.mean, ssize, error) {
+     error.val <- rnorm(sd = error, n = ssize)
+     sample.from.control <- sample(list.skew, prob = freq.skew,
+         size = ssize, replace = TRUE)
+     if (bot.mean == 0) {
+         poisson.dens <- rep(0, 40)
+         poisson.dens[1] = 1
+     }
+     else {
+         poisson.dens <- dpois(lambda = bot.mean, x = c(1:40))
+         poisson.dens <- poisson.dens/sum(poisson.dens)
+     }
```

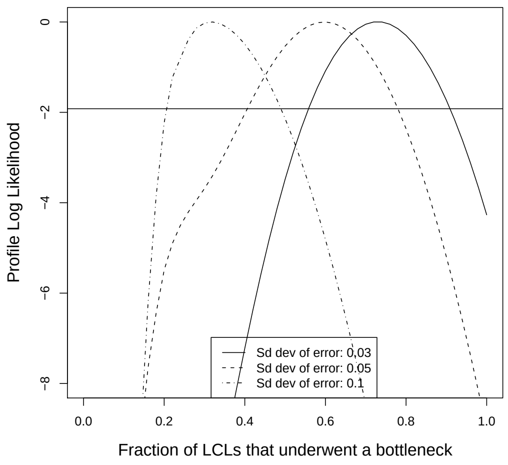

Figure 2: Likelihood curve for the fraction of cells f that underwent a bottleneck. We consider three values for the standard error in the measurement of the skew in X inactivation (standard error of 2%, 5% and 10%). See Supplemental Information for a detailed description.

```
+     bot <- sample(replace = TRUE, prob = poisson.dens, size = ssize,
+         x = c(1:40))
+     my.binom <- rbinom(n = ssize, size = bot, prob = sample.from.control)/bot
+     simulated <- ifelse(runif(n = ssize) < freq, my.binom, sample.from.control) +
+         error.val
+     simulated <- ifelse(simulated < 0, 0, simulated)
+     simulated <- ifelse(simulated > 1, 1, simulated)
+     simulated <- round(simulated * 100)
+     simulated <- pmax(simulated, 100 - simulated)
+     simul <- hist(simulated, plot = FALSE, breaks = bp)
+     pdf("fig/simulated.pdf")
+     par(cex.lab = 1.3, cex.main = 1.5)
+     names <- c("50-55", "55-60", "60-65", "65-70", "70-75", "75-80",
+         "80-85", "85-90", "90-95", "95-100")
+     barplot(rbind(100 * BBC$counts/sum(BBC$counts), 100 * T1D$counts/sum(T1D$counts),
+         100 * simul$counts/sum(simul$counts)), beside = TRUE,
+         names = names, cex.names = 0.7, ylim = c(0, 35), col = c("white",
+             "grey", "black"), ylab = "Percentages", xlab = "Measured skew in X inactivation")
+     title("Simulated distribution of XCI")
+     legend(legend = c("1958 BBC transformed cell lines", "T1D transformed cell lines",
+         "Simulated cell line data"), "topright", fill = c("white",
+         "grey", "black"))
+     dev.off()
+ }
```

We can run the simulations using the following code. Results are shown in Figure 3.

```
> simulate(freq = 0.22, bot.mean = 0, ssize = 5000, error = 0.05)
```

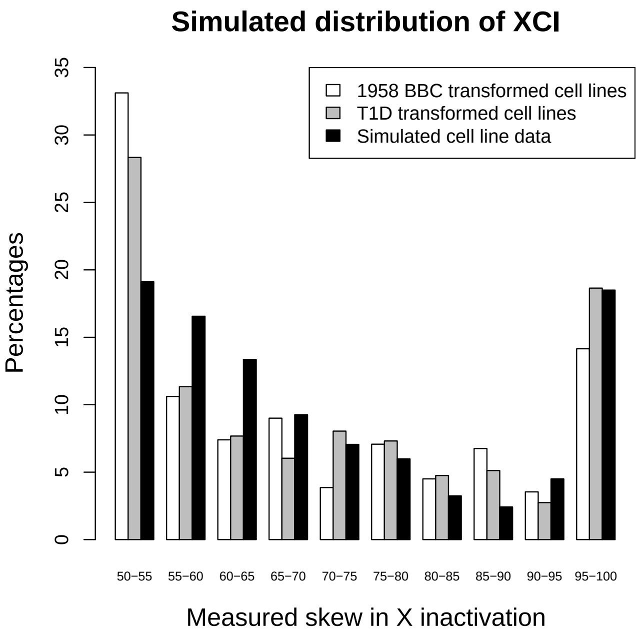

Figure 3: Simulation study comparing the XCI between our best fitting scenario and both sets of cell line (1958 British Birth Cohort and T1D samples).

```
null device
          1
```
