# Supplementary material for: Extreme Clonality in Lymphoblastoid Cell Lines with Implications for Allele Specific Expression Analyses
Source: PLoS One. 2008 Aug 13;3(8):e2966. doi: 10.1371/journal.pone.0002966 (PMC2494943; doi:10.1371/journal.pone.0002966)
Supplement: Protocol S1 — Protocol for cell line transformation. (0.07 MB PDF) [file pone.0002966.s003.pdf]

Extreme clonality in lymphoblastoid cell lines with implications for allele specific expression analyses

Vincent Plagnol, Elif Uz, Chris Wallace, Helen Stevens,
David Clayton, Tayfun Ozcelik and John A. Todd

Protocol for cell line transformation

Type 1 diabetes samples

The cell line transformation process requires collection of fresh blood samples. 89% of blood samples used in this study were less than 4 days old post venepuncture with 6-9 ml, optimal, total blood volume being obtained, wherever possible, to ensure that sufficient cells can be isolated from the blood for immortalisation. For 84% of samples the starting volume was greater than 6 ml blood although less than 6 ml of blood was drawn in some instances due to the young age of some patients.

Peripheral Blood Lymphocyte (PBL) cells were isolated from the whole blood on receipt of the sample using *Accuspin* system Histopaque 1077 (when PBL isolation occurred within 3 days of collection) or Lympholyte (*CedarLane laboratories*). PBLs were then frozen until required for transformation. Approximately 4×10^6 PBLs are required to produce a successful transformation so resulting PBL pellets were split and frozen in 1-3 aliquots according to size. After rapid thawing and washing with Hanks' balanced salt solution (HPSS), the cells were re-suspended with Lymphocytic Cell Line (LCL) media (Dutch modified RPMI with 20% FBS, 1% Pen/Strep, 1% L-Glutamine and 0.1% Gentamicine) and EBV supernatant. Cells were left for 5-7 days at (37°C, 5% CO2) to take up the virus and proliferate. During this time clumps of cells become visible in the culture, as cell growth proliferates post infection. Upon reaching confluency the line is slowly expanded as the cells are fed with small volumes of LCL media. Feeding continues until 100 ml of confluent cell line is obtained. At this stage the line is split into two. Half of the cells are used to create a frozen back up of the cell line and the other half of the cells are pelleted for subsequent DNA extraction. Washing with LCL media or enriched LCL media ($>$ 20% FBS) and re-infection with the EBV virus can be used to encourage a slow growing cell line. DNA was extracted using an organic chloroform extraction protocol [1]

British 1958 Birth Cohort samples

This transformation protocol is similar to the protocol used at ECCAC (`http://www.ecacc.org.uk/`). PBLs were isolated from 9 ml blood samples and frozen in two pellets. Only one of the two pellets was used for transformation (hence from a starting volume of 4.5 ml blood). Cells were split between 1, 2, 4 or 8 wells of a microtitre cell plate depending on cell count and were infected with EBV. Cells were plated and expanded with the help of a robot. Cells were incubated (37C, 5% CO2) and over time proliferate and expand. Confluent wells were pooled together (4 wells become 2, 2 wells become 1 etc..) and fed with cell media which slowly expanded the line. Finally cells from the whole plate were pooled into a single tube and are spun down to create the frozen back up of the cell line. A fraction of the pooled cells were further expanded to create a pellet for subsequent DNA extraction.

References

[1] Ueda H, Howson JM, Esposito L, Heward J, Snook H, et al. (2003) Association of the T-cell regulatory gene CTLA4 with susceptibility to autoimmune disease. Nature 423:506–511.
